# The Rubber Patents

**Published:** 1880-12

**Authors:** 


### THE RUBBER PATENTS.

*The Dental Jairus* states that the Goodyear Dental Vulcanite Co., have already made applications for a re-issue of their patent which expires by limitation in June, 1881. We think the *Dental Jairus* is mistaken. The only way in which the Goodyear Dental Vulcanite Co., can procure an extension of the Cummings patent is by special act of Congress, and we are assured that no such application has yet been presented to the Patent Committees of either the Senate or House of Representatives. We shall endeavor to keep the profession posted as to any move in that direction.—*The Dental Advertiser, Oct.* 1880.

